# Spasmolytic activity of *Aquilariae Lignum Resinatum* extract on gastrointestinal motility involves muscarinic receptors, calcium channels and NO release

**DOI:** 10.1080/13880209.2018.1492000

**Published:** 2018-12-05

**Authors:** Huimin Li, Yanfei Qu, Jiawei Zhang, Jingze Zhang, Wenyuan Gao

**Affiliations:** a Department of Pharmacy, Special Drugs R&D Center of People’s Armed Police Forces, Logistics University of Chinese People’s Armed Police Forces, Tianjin, China;; b School of Pharmaceutical Science and Technology, Tianjin University, Tianjin, China

**Keywords:** Cholinergic, Ca^2+^ channels, nitric oxide, interstitial cells of Cajal, Wei-Chang-An pill, neostigmine

## Abstract

**Context:** *Aquilariae Lignum Resinatum* (ALR), the dry rhizome of *Aquilaria agallocha* R. (Thymelaeaeeae), has been widely used to treat emesis, stomachache and gastrointestinal dysfunction.

**Objective:** This study evaluates the effects of ALR methanol extract on gastrointestinal motility (GIM) and possible mechanisms of the action involved.

**Materials and methods:** *In vivo,* the study evaluated the effects of ALR (200–800 mg/kg) on gastric emptying and small intestinal motility in normal and neostigmine-induced adult KM mice. The *in vitro* effects of ALR (0.2–1.6 mg/mL) on GIM were performed on isolated jejunum of Wistar rats, pretreated with acetylcholine (ACh), KCl, CaCl_2_, and pre-incubation with l-NAME (a selective inhibitor of the nitric oxide synthase).

**Results:** *In vivo*, ALR (800 mg/kg) decreased gastric emptying (70.82 ± 9.81%, *p* < 0.01, compared with neostigmine group 91.40 ± 7.81%), small intestinal transit (42.82 ± 3.82%, *p* < 0.01, compared with neostigmine group 85.53 ± 5.57%). *In vitro*, ALR concentration dependently decreased the contractions induced by ACh (10^−5^ M) and KCl (60 mM) with respective EC_50_ values of 0.35 and 0.32 mg/mL. The Ca^2+^ concentration–response curves were shifted by ALR to the right, similar to that caused by verapamil (the positive). The spasmolytic activity of ALR was inhibited by pre-incubation with l-NAME.

**Discussion and conclusions:** ALR played a spasmolytic role in GIM, which is probably mediated through inhibition of muscarinic receptors, blockade of Ca^2+^ influx and NO release. This is the first study presenting a comprehensive description of the effects of ALR on GIM.

## Introduction

Functional gastrointestinal (GI) diseases such as functional dyspepsia, irritable bowel syndrome, gastroesophageal reflux, as well as other symptoms (e.g., abdominal pain, nausea, vomiting, diarrhoea, constipation, etc.) are common and varied pathological illnesses in the GI tract (Chang 2004; Defrees and Bailey 2017; Vakil et al. 2017; Pandit et al. 2018). These diseases have adverse effects on the quality of people’s lives and greatly increase health-care costs. The physiological mechanisms of above symptoms are critically related to the gastrointestinal motility (GIM) disorders (Kimura and Sumiyoshi 2012). Among various available drugs treatment for these diseases, chemical drugs are common, while a large number of adverse effects caused by them highlight the need for safer and more effective agents. Currently, with a growing interest in natural medicine, it is found that herbal medicines are composed of multiple biologically active compounds on multiple pathophysiological targets. Several studies have been undertaken to provide scientific proof to justify the medicinal use of various plants in the treatment for GI tract diseases (Hu et al. 2009; Lam et al. 2010; Mehmood et al. 2011; Neamsuvan and Ruangrit 2017).

*Aquilaria agallocha* R. (Thymelaeaeeae), recorded in the Chinese Pharmacopoeia (Committee of National Pharmacopoeia 2015), is an economically important tree because of its resinous heartwood. The plant is widely used in perfumery, medicine and agarbathi (incense sticks) industries (Yang et al. 2014). *Aquilariae Lignum Resinatum* (ALR) is one of the important drugs for promoting qi circulation and relieving pain in the clinical practice of Traditional Chinese Medicine. Traditionally, it is suggested in prescriptions or used as an herb for the treatment of GI dysfunction such as peptic ulcer, diarrhoea, nausea and vomiting. The plant is also boiled as tea or soaked in wine by the indigenous people, which are thought to be beneficial for the GI tract (Kakino et al. 2010). Phytochemical investigations have shown ALR possesses sesquiterpenes, 2-(2-phenylethyl) chromone derivatives, flavonoids, fatty acids, etc. Pharmacological research demonstrated the anaesthetic, spasmolytic, and analgesic activities of sesquiterpenes, anti-inflammatory and antispasmodic activities of flavonoids, sedative action of baimuxinic acid, antiallergic effect of 2-(2-phenylethyl) chromone, and antibacterial activities of volatile components (Kang et al. 2003; Boussaada et al. 2008; Li et al. 2014). Earlier studies have also shown that ALR effectively regulates on the GI tract (Zhou 1988; Tian et al. 2009). Even so, there is no study focusing on the antispasmodic activity of ALR on the GI tract and scarce data are available about the possible mechanisms, especially the role of gaseous mediators.

As a consequence, the present study was undertaken to elucidate the effects of ALR on GI tract using *in vivo* and *in vitro* tests. In order to understand the mechanisms, some of the relevant mediators involved in the action of ALR were identified using receptor, ion channel binding assays and inhibitors of gas transmitter *in vitro* tests. Furthermore, the chemical analysis of ALR was performed by UHPLC-Q-TOF-MS to ensure its chemical consistency. All these results may provide imperative pharmacological evidence for its folk and clinical uses in GIM diseases.

## Materials and methods

### Plant materials and preparation of extract

*Aquilariae Lignum Resinatum* was provided by Tianjin Lerentang Pharmaceutical Factory and identified by Professor Wenyuan Gao from the School of Pharmaceutical Science and Technology, Tianjin University, China. All the voucher specimens (Voucher No. CX1403105) were conserved at the School of Pharmaceutical Science and Technology, Tianjin University.

ALR (100 g) was powdered and extracted with 1 L of methanol for 1 h, three times. The combined methanol extracts were concentrated using a rotary evaporator at reduced pressure and the crude extract was obtained with a yield of 12.18% (w/w). ALR was suspended in 0.5% carboxymethyl cellulose (CMC) for *in vivo* experiments. For *in vitro* tests, ALR was first ground with Tween-80, the solution was diluted with distilled water so that the final concentration of Tween-80 was 1% in the vehicle. Vehicles used had no effect in control experiments.

### Drugs and reagents

ACh was purchased from the National Institute for Control of Pharmaceutical and Biological Products (Beijing, China). Neostigmine methylsulphate injection (2 mL:1 mg) and verapamil were donated by Affiliated Hospital of Logistics College of Chinese People’s Armed Police Forces. l-NAME and PAG were obtained from Sigma-Aldrich Co. (St. Louis, MO). Acetonitrile, methanol and formic acid were purchased from Merck (Darmstadt, Germany). HPLC grade water was obtained from a Milli-Q Reagent Water System (Millipore, Burlington, MA). Wei-Chang-An pill (WCA) was obtained from Tianjin Lerentang Pharmaceutical Factory (Tianjin, China). All the other reagents were obtained from Tianjin Jiangtian Chemical Reagent Science and Technology Co., Ltd. (Tianjin, China). These reagents were of analytical grade. Solutions of all drugs and reagents were dissolved with saline or distilled water, and they were freshly prepared on the day of experiments.

### Animals

Adult KM mice (18–22 g) and Wistar rats (280–330 g) were obtained from the Laboratory Animal Center of Health Science, Peking University, Beijing, China (License No. SCXK (Jin)-2009-003). All animals were housed in an environmentally (*t* = 25 ± 1 °C) and air humidity (60%) controlled room with a 12 h light/dark cycle. They have free accessed to water, but were fasted for 24 h before experiments. The animals were cared for in accordance with the Guide to the Care and Use of Experimental Animals.

### Chemical analysis of ALR by UHPLC-Q-TOF-MS

The chemical analysis was performed on an Agilent 1290 UHPLC system connected with Agilent 6520 Q-TOF mass spectrometer via an ESI interface (Agilent Technologies, Palo Alto, CA). Samples were separated on a reversed phase C18 analytical column (2.1 mm × 100 mm, 1.7 μm). The mobile phase consisted of water–formic acid (100:0.5, v/v) (eluent A) and acetonitrile–methanol (50:8) (eluent B), the linear gradient as follows: 0–40 min, 5–100% B; 40–50 min, 100% B; flow rate, 0.5 mL/min. For MS detection, operating conditions were as follows: drying gas (N_2_) flowrate, 10.0 L/min; drying gas temperature, 360 °C; nebulizer gas pressure, 40 psig; capillary voltage, 4.5 kV; fragmentor voltage, 175 V; skimmer voltage, 65 V; octopole radio frequency voltage, 750 V. The MS spectra were acquired under the positive ion mode. The collision energy was set at 15–40 V and the accurate mass spectra were recorded across the range 100–1700 *m*/*z*.

### *In vivo* study

#### Gastric emptying and small intestinal transit in normal mice

Sixty mice were randomly divided into six groups of 10 mice for each group. Vehicle control group (0.5% CMC, 10 mL/kg), ALR-treated groups (200, 400 and 800 mg/kg) and positive control group (WCA, 1600 mg/kg) were intragastrically administered in a volume of 0.2 mL/20 g for seven days. Gastric emptying and small intestinal transit rate in 24 h fasted mice were measured using the phenol red method (Qu et al. 2014). A test meal containing phenol red (0.5 mg/mL) was orally administered in a volume of 0.3 mL to mice 30 min later after the last administration. Mice were killed by cervical dislocation after 20 min. The stomach and intestine were immediately removed. The amount of phenol red retained in the stomach, the total length of the small intestine and the distance travelled by phenol red were measured.

The amount of phenol red maintained in the stomach was determined by the following methods. The stomach was cut into several pieces and homogenized in 25 mL of 0.1 M NaOH. The suspension was allowed to stand at room temperature for 1 h, and 1 mL of trichloroacetic acid (33% w/v) to 8 mL of the supernatant was added to remove protein. The mixture was centrifuged at 3200×*g* for 10 min. 2 M NaOH (1 mL) was added to 4 mL of the supernatant. The absorbance of the sample was read at 560 nm by with the 754 spectrophotometer. In addition, another 10 mice were sacrificed immediately after administration of the test meal, and its absorbance of phenol red is regarded as a standard. Gastric emptying rate was calculated according to the formula: Gastric emptying rate (%) = 1 − (amount of phenol red recovered from test stomach/average amount of phenol red recovered from standard stomach × 100).

After the excision of the stomach, the length of the whole small intestine was measured from the pyloric sphincter to the ileocecal junction. The intestine was exactly localized by a drop of 0.1 M NaOH to measure the distance travelled by the phenol red. Small intestinal transit rate was expressed as the distance travelled by the test meal relative to the total length of the small intestine (Mulè et al. 2010). Intestinal transit rate was calculated by the following formula: Intestinal transit rate (%) = the distance travelled by the phenol red/the total length of the small intestine × 100.

#### Gastric emptying and small intestinal transit in neostigmine-induced mice

Fifty animals were randomly divided into five groups, including vehicle control group (0.5% CMC, 10 mL/kg), positive control group (WCA, 1600 mg/kg) or ALR-treated groups (200, 400 and 800 mg/kg). They were administered intragastrically for 30 min prior to intraperitoneal injection of neostigmine (0.1 mg/kg). Phenol red was administered 15 min later and 20 min thereafter, the mice were sacrificed. The gastric emptying and intestinal transit rate were calculated as the method described on the former part.

### *In vitro* study

#### Isolated jejunum preparations

Wistar rats were killed by cervical dislocation after 24 h starvation. The jejunum was removed, flushed, then put into the 0 °C Tyrode’s solution (composition (mM) NaCl 136.9, CaCl_2_ 1.8, KCl 2.7, MgCl_2_ 1.1, NaHCO_3_ 11.9, NaH_2_PO_4_ 0.4, and glucose 5.6, pH 7.4). The jejunum tissue was prepared with approximately 2 cm length, and placed in a 10 mL organ bath with Tyrode’s solution, continuously bubbled with a mixture of O_2_ and CO_2_ (19:1), and maintained at 37 °C. The resting tension was subjected to 1.5 g and it took 30 min to equilibrate before any drug added.

#### The effects of ALR on spontaneous, Ach and KCl-induced contractions of rat-isolated jejunum

Rat-isolated jejunum was suspended in Tyrode’s solution and equilibrated for 30 min. Different concentrations of ALR (0.2, 0.4, 0.6, 0.8, 1.2, 1.4 and 1.6 mg/mL) were added sequentially into the bath to examine the effects of ALR on the spontaneous contractions of jejunum strips. In order to investigate prokinetic mechanism preliminarily, the effects of ALR on the contractions of jejunum strips induced by pretreatment with Ach (10^−5^ M) and KCl (60 mM) were tested (Rahman et al. 2017).

#### The effects of ALR on CaCl_2_-induced contractions of rat-isolated jejunum

To assess whether the spasmolytic activity of ALR was mediated through the blockade of calcium-channel, high K^+^ (60 mM) was used to depolarize the preparations. The jejunum strips were initially stabilized in normal Tyrode’s solution and then replaced with a high-K^+^ (60 mM) and Ca^2+^-free solution (composition (mM) KCl 60, NaCl 91.04, MgCl_2_ 1.05, NaHCO_3_ 11.90, NaH_2_PO_4_ 0.42, glucose 5.55 and EDTA 0.1) for 30 min to remove Ca^2+^ in the tissues (Bashir et al. 2011; Alam et al. 2016). CaCl_2_ (3 × 10^−5^ to 3 × 10^−3^ M) was added exogenously in a cumulative manner to establish CaCl_2_ concentration–response curves. The concentration–response curves of CaCl_2_ were constructed in the absence and the presence of ALR (0.2, 0.4, 0.8 and 1.6 mg/mL) or verapamil (0.025 mM, positive control). The contraction induced by 3 × 10^−3^ M CaCl_2_ in the absence of ALR and verapamil was considered as 100%.

#### Role of NO and H_2_S on ALR-mediated relaxation of rat-isolated jejunum

To analyse the role of gaseous mediator on ALR-mediated relaxation on the contractions of rat-isolated jejunum, inhibitors of NO and H_2_S synthetase were used. l-NAME (a NO synthase inhibitor, 10^−4^ M) and PAG (a cystathionine-γ-lyase inhibitor, 10^−5^ M) were used to pre-incubate the jejunum tissues for 20 min. Then, the contraction of rat jejunum strips was induced by ACh (10^−5^ M). ALR (0.2, 0.4, 0.8 and 1.6 mg/mL) was added sequentially to examine the effects of endogenous NO and H_2_S on ALR-mediated relaxation. Incubation in the absence of inhibitors was considered as a control group.

### Data analysis

SPSS17.0 system (Chicago, IL) was used to analyse the data. All data were expressed as mean ± standard error of mean (S.E.M.) or percentage. Differences among groups were analysed by a one-way analysis of variance (ANOVA) followed by Dunnett’s test. *p* Value less than or equal to 0.05 was noted as statistically significant.

## Results and discussion

### Chemical analysis of ALR by UHPLC-Q-TOF-MS

**Figure 1.**
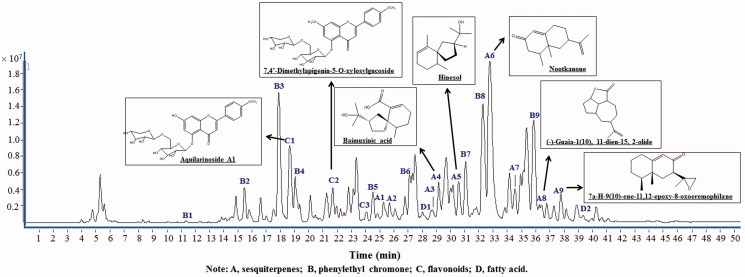
The UHPLC-Q-TOF-MS chromatograms of ALR methanol extracts.

To characterize the chemical constituents in ALR, an UHPLC-Q-TOF-MS method was established. Altogether, a total of 23 compounds were tentatively identified and characterized in ALR, including nine sesquiterpenes (A1–A9), ten 2-(2-phenylethyl) chromone (B1–B9), three flavonoids (C1–C3) and two fatty acids (D1, D2) (Figure 1, Table 1). All compounds were tentatively characterized based on matching the empirical molecular formula with those of the reported compounds, UV spectra, retention time and MS spectra as well. The mass errors for molecular ions of all identified compounds were within ±0.01 ppm. These 23 identified compounds represented the major components in ALR. The majority of them had been reported to be main bioactive constituents of the plant.

**Table 1.** Characterization of compounds of ALR by UHPLC-Q-TOF-MS.

No.	*t*_R_ (min)	Identification	Formula	MW calculated	Quasi-molecular	Error (ppm)
B1	11.325	6,7-Dihydroxy-2-(2-phenylethyl)-5,6,7,8-tetrahydrochromoe	C_17_H_18_O_4_	286.1205	287.1278 (M + H)^+^	–3.50
B2	15.452	5,6,7,8-Tetrahydroxy-2-(3-hydroxy-4-methoxyphenethyl)-5,6,7,8-tetrahydro-4H-chromen-4-one	C_18_H_20_O_8_	364.1158	387.1050 (M + Na)^+^	–6.85
B3	17.971	(5S*,6R*,7S*)-5,6,7-Trihydroxy-2-(3-hydroxy-4-methoxyphenethyl)-5,6,7,8-tetrahydro-4H-chromen-4-one	C_18_H_21_O_7_	349.1209	372.1179 (M + Na)^+^	–6.42
C1	18.700	Aquilarinoside A1	C_28_H_32_O_15_	608.1741	609.1834 (M + H)^+^	–3.95
B4	19.031	5,6,7,8-Tetrahydro-5β,6β,7α,8β-tetrahydroxy-2-(2-phenylethyl)chromone	C_17_H_19_O_6_	319.1103	342.1074 (M + Na)^+^	–3.92
C2	21.932	7,4′-Dimethylapigenin-5-*O*-xylosylglucoside	C_28_H_32_O_14_	592.1792	593.1865 (M + H)^+^	–4.97
C3	23.871	7,4′-Dimethyl-5-*O*-glucosideflavonoide	C_23_H_24_O_10_	460.1369	461.1442 (M + H)^+^	–4.37
B5	24.500	5-Hydroxy-6-methoxy-2-[2-(3-hydroxy-4-methoxyphenyl)ethyl]chromone	C_19_H_18_O_6_	342.11.3	343.1176 (M + H)^+^	–3.95
A1	25.395	*cis*-1,10-Epoxyguaia-11-en-2-ol	C_15_H_24_O_2_	236.1776	237.1749 (M + H)^+^	–4.42
A2	25.895	Agarofuran-4-hydroxylbaimuxinol	C_15_H_26_O_3_	254.1882	259.1669 (M + Na)^+^ [–H_2_O]	0.93
B6	27.102	6,7-Dimethoxy-2-[2-(3-methoxyphenyl)ethyl]chromone; 5,8-Dihydroxy-2-[2-(4-methoxyphenyl)ethyl]-chromone	C_18_H_16_O_5_	312.0998	313.1071 (M + H)^+^	–4.62
D1	28.362	Dodecanoic acid	C_12_H_24_O_2_	200.1776	223.1669 (M + Na)^+^	–2.20
A3	28.941	Epoxybulnesene	C_15_H_24_O	220.1827	203.1794 (M + H)^+^ [–H_2_O]	–3.96
A4	29.273	Baimuxinic acid	C_15_H_24_O_3_	252.1725	275.1618 (M + H)^+^	–2.28
A5	30.751	Hinesol	C_15_H_26_O	222.1984	205.1951 (M + H)^+^ [H_2_O]	0.66
B7	31.046	7-Hydroxy-2-(2-phenylethyl)chromone; 6-Hydroxy-2-(2-phenylethyl)chromone	C_17_H_15_O_3_	267.0943	290.0613 (M + Na)^+^	3.21
B8	32.256	6,7-Dimethoxy-2-[2-(4′-methoxyphenyl)ethyl]chromone	C_20_H_20_O_5_	340.1311	363.1203 (M + Na)^+^	–5.00
A6	32.919	Nootkanone	C_15_H_22_O	218.1671	209.1743 (M + H)^+^	–1.78
A7	34.808	β-Vatirenene	C_15_H_22_	202.1722	203.1794 (M + H)^+^	–2.06
B9	34.924	2-(2-Phenyleyhyl)chromone	C_17_H_14_O_2_	250.1994	251.1067 (M + H)^+^	–4.54
A8	36.382	(–)-Guaia-1(10),11-dien-15,2-olide	C_15_H_20_O_2_	232.1463	255.1356 (M + Na)^+^	–5.12
A9	37.626	7b-H-9(10)-ene-11,12-Epoxy-8-oxoeremophilane; 7a-H-9(10)-ene-11,12-epoxy-8-oxoeremophilane	C_15_H_22_O_2_	234.1620	235.1693 (M + H)^+^	–3.36
D2	39.150	*n*-Hexadecanoic acid	C_16_H_32_O_2_	256.2402	279.2295 (M + Na)^+^	–4.58

*t*_R_: retention time; A: sesquiterpenes; B: phenylethyl chromone; C: flavonoids; D: fatty acid.

### Gastric emptying and small intestinal transit in normal and neostigmine-induced mice *in vivo* study

*In vivo* study, the gastric emptying rate was 62.93 ± 8.74% in normal conditions. ALR at different doses treatment had a little effect on gastric emptying compared with normal mice (*p* > 0.05). In groups of ALR (200, 400 and 800 mg/kg), the gastric emptying rates were 66.78 ± 7.25%, 64.34 ± 5.47% and 59.74 ± 9.81%, respectively (Table 2). However, the gastric emptying was significantly promoted by neostigmine in normal mice (91.40 ± 7.81%). Administration of ALR inhibited the high gastric emptying induced by neostigmine, especially in the dose of 800 mg/kg (70.82 ± 9.81%, *p* < 0.01, compared with neostigmine group) (Table 3).

**Table 2.** Effects of different doses of ALR on gastric emptying and small intestinal transit in normal mice (*n* = 10).

Treatment group	Dosage (mg/kg)	Gastric emptying (%)	Intestinal transit (%)
Normal	CMC-Na	62.93 ± 8.74	64.16 ± 7.00
WCA	1600	45.85 ± 5.63**	70.58 ± 4.07*
ALR	200	66.78 ± 9.70	52.78 ± 6.16*
ALR	400	64.24 ± 6.96	48.07 ± 5.96*
ALR	800	59.74 ± 9.81	42.82 ± 3.82**

ANOVA followed by Dunnett’s multiple comparison test.

Compared with neostigmine mice:

*p < 0.05.

**p < 0.01.

**Table 3.** Effects of different doses of ALR on gastric emptying and small intestinal transit in neostigmine-induced mice (n = 10).

Treatment group	Dosage (mg/kg)	Gastric emptying (%)	Intestinal transit (%)
Neostigmine	CMC-Na	91.40 ± 7.81	85.53 ± 5.57
WCA	1600	65.93 ± 6.65**	63.58 ± 3.40**
ALR	200	78.11 ± 9.70*	77.03 ± 6.16*
ALR	400	73.22 ± 6.96**	73.91 ± 5.96*
ALR	800	70.82 ± 9.81**	69.60 ± 3.82**

ANOVA followed by Dunnett’s multiple comparison test.

Compared with neostigmine mice:

*p < 0.05.

**p < 0.01.

In small intestinal transit tests, an evident inhibitory effect of ALR on intestinal transit both in normal and neostigmine-treated mice was observed. As indicated in Table 2, the small intestinal transit was dose dependently suppressed after ALR treatment in normal mice. The strongest inhibitory effect of ALR on small intestinal transit was 42.82 ± 3.82% at the dose of 800 mg/kg (*p* < 0.01). In neostigmine-treated mice, neostigmine obviously accelerated the propulsion of the phenol red in the small intestine (85.53 ± 5.57%). The stimulated intestinal transit induced by neostigmine was significantly attenuated by ALR in a dose-dependent manner, with the values of 77.02 ± 6.16%, 73.91 ± 5.96% and 69.60 ± 3.82% (*p* < 0.05), respectively. The strongest inhibitory activity of ALR was comparable to the positive control (WCA, 63.58 ± 3.40%, *p* < 0.01) (Table 3).

### Inhibitory effects of ALR on spontaneous, ACh and KCl-induced contractions of rat-isolated jejunum *in vitro* study

**Figure 2.**
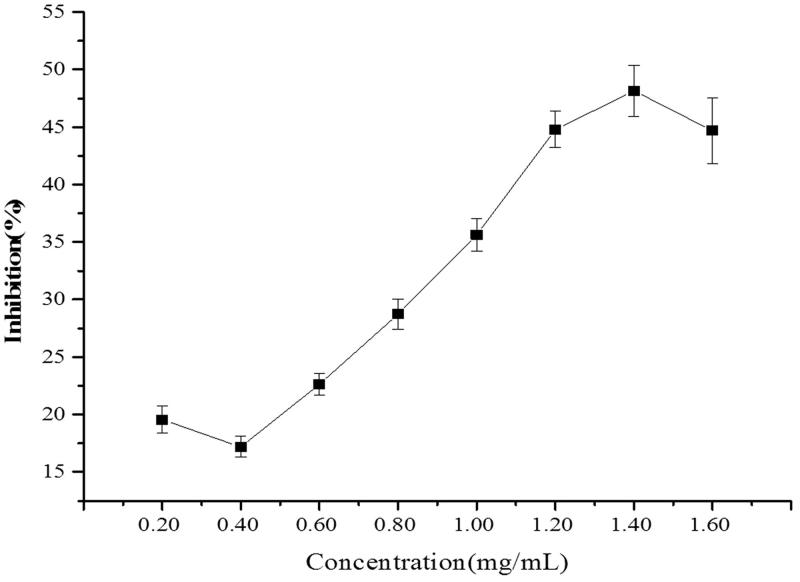
Inhibitory effects of different concentrations of methanol extract of ALR on the spontaneous contraction of rat-isolated jejunum (*n* = 6).

**Figure 3.**
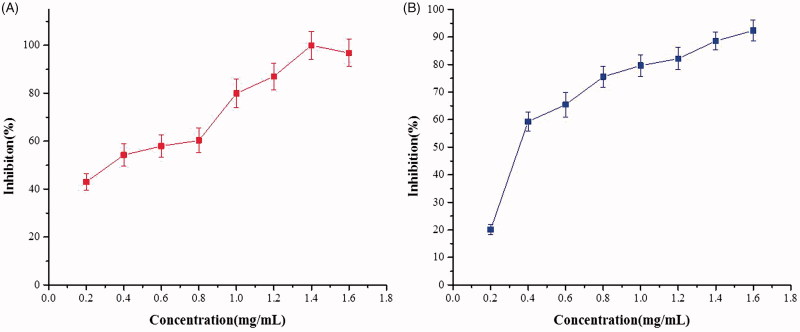
Inhibitory effects of different concentrations of methanol extract of ALR on ACh and KCl-induced contractions of rat-isolated jejunum (*n* = 6). (A) ACh-contractions; (B) KCl-induced contractions.

ALR exhibited an inhibitory effect on the spontaneous contractions of isolated rat jejunum in a concentration-dependent manner ranging from 0.2 to 1.6 mg/mL. The maximum inhibition was up to 48.14% (Figure 2). ALR also concentration dependently decreased the contractions induced by ACh (10^−5^ M) and KCl (60 mM) with respective EC_50_ values of 0.35 mg/mL and 0.32 mg/mL. The strongest inhibitory effects were 100.00 ± 5.92% and 92.38 ± 3.95%, respectively (Figure 3).

### The effects of ALR on CaCl_2_-induced contractions of rat-isolated jejunum

**Figure 4.**
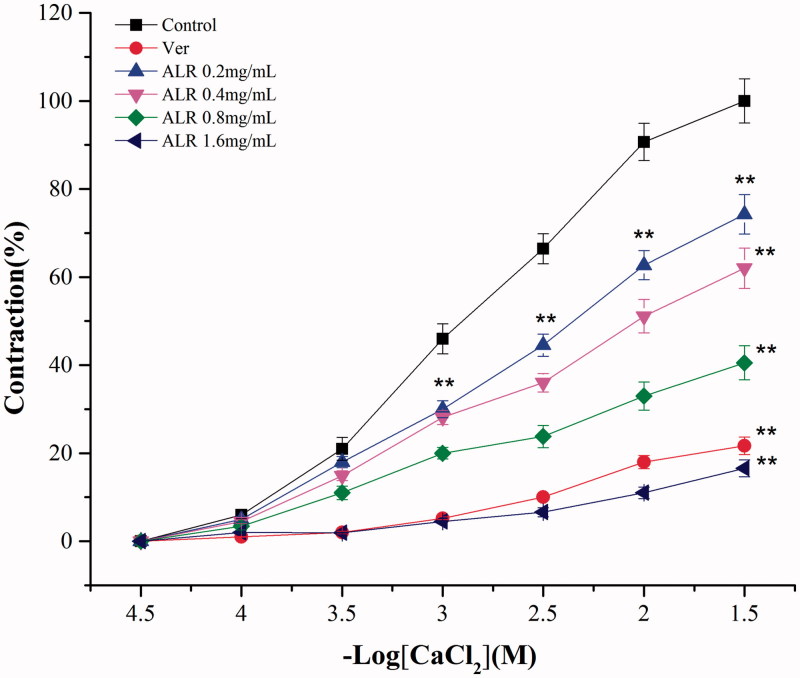
Concentration–response curves of CaCl_2_ on rat-isolated jejunum in the absence and in the presence of ALR and verapamil (*n* = 6). Compared with vehicle control group: **p < 0.01. ANOVA followed by Dunnett’s multiple comparison test.

As the results shown in Figure 4, the inhibitory effects of ALR on CaCl_2_-induced contractions were increased in a concentration-dependent manner. The concentration–response curves were shifted to the right by ALR. The maximum contraction induced by 3 × 10^−3^ M CaCl_2_ was reduced to 74.25%, 62.00%, 40.54% and 16.56% after ALR (0.2, 0.4, 0.8 and 1.6 mg/mL) treatment. The positive control group (verapamil, 0.025 mM) reduced the maximum contraction to 21.68%. The curve of CaCl_2_ concentration–response in the presence of ALR (1.6 mg/mL) was similar to that of verapamil. They all had significant difference compared with vehicle control (*p* < 0.01).

### Role of NO and H_2_S on ALR-mediated relaxation of rat-isolated jejunum

**Figure 5.**
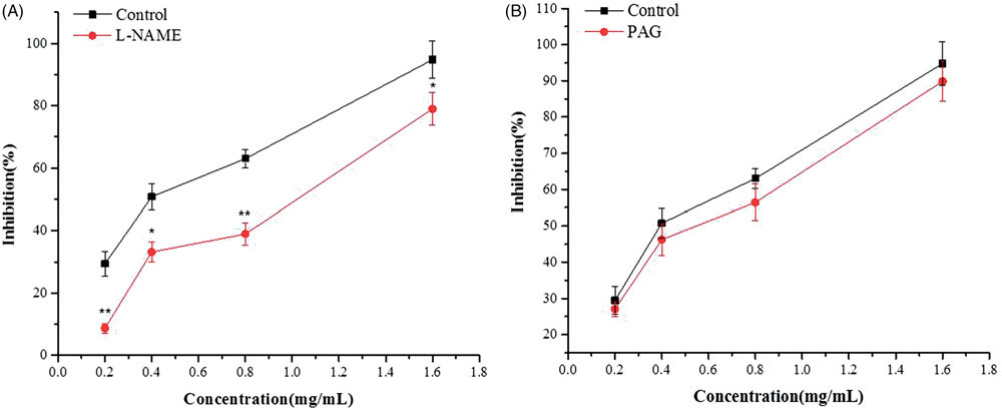
Effects of different inhibitors on ALR-induced relaxation in rat-isolated jejunum. (A) Effect of l-NAME 10^–4^ M on ALR-induced relaxation. (B) Effect of PAG 10^–5^ M on ALR-induced relaxation. Compared with vehicle control group: **p < 0.01. ANOVA followed by Dunnett’s multiple comparison test.

ALR-mediated relaxation of rat-isolated jejunum was significantly reduced by pretreatment with l-NAME (10^−4^ M) in a concentration-dependent manner (*p* < 0.05) (Figure 5(A)). However, pre-incubation with PAG (10^−5^ M) did not produce distinct change in the relaxant effect on the relaxation induced by ALR (*p* > 0.05) (Figure 5(B)).

Numerous studies have validated the traditional use of medicinal plants of the GIM diseases by investigating the biological activity of such plants, which have antispasmodic effects, delay intestinal transit, inhibit gut motility, stimulate water adsorption or reduce electrolyte secretion (Guo et al. 2014). Several previous studies also supported the hypothesis that GIM was not only controlled by GI peptides (gastrin and ghrelin) or intracellular Ca^2+^, but also by various neurotransmitters such as ACh, dopamine and 5-hydroxytryptamine (5-HT) (Chen et al. 2010). Besides, the gaseous mediator has been regarded as another important factor that mediates GIM in either physiological or certain pathophysiologic states (Wirthlin et al. 1996; Takahashi 2003; Martinze-Cutillas et al. 2015). In our study, we found that ALR presented inhibitory effects on the GIM, and mediated possibly through the combination of anticholinergic, Ca^2+^ antagonist and gasotransmitters.

Gastric emptying and small intestinal transit were evaluated with *in vivo* tests because these states relatively consistent with the physiological foundation of GI diseases. The actions of gastric emptying and small intestinal transit were stimulated by releasing endogenous ACh (Kimura and Sumiyoshi 2011). In this study, an interesting phenomenon was found that ALR had a little effect on gastric emptying, but exhibited an inhibitory effect on intestinal transit in normal mice. This might be due to the different regulatory mechanisms of stomach and small intestine in normal condition. The pathway of gastric emptying was complex, which was involved in cholinergic excitation or direct action on the pyloric sphincter (Amira et al. 2005). Additionally, in the present study, the GI hyperfunction animal model was established by intraperitoneal injection of neostigmine *in vivo* tests. Neostigmine is an acetylcholinesterase (AChE) inhibitor that could increase the cholinergic activity in gut wall. The activated cholinergic activity results in the contractile action. The drug is usually used to mimic the general mechanism of action of many prokinetic drugs (Cellini et al. 2011). Neostigmine treatment induced an evident increase of gastric emptying and intestinal transit, which confirms the importance of the cholinergic pathway in controlling the flow from stomach to intestine. In this study, an inhibitory effect of ALR treatment was observed both on gastric emptying and intestinal transit in neostigmine-treated mice. The results indicated that ALR held possible protective effects against neostigmine-induced GI excitatory movement. The inhibitory action of ALR on GIM might be related to the release of ACh from autonomic neurons.

As intestinal transit inhibited by ALR in both normal and neostigmine-induced mice, we supposed that the activity was attributed to relaxing the contractions of the smooth muscle layers. Thus, we investigated the effects of ALR on the contractile activity of the smooth muscle layers with *in vitro* tests. Spasmolytic activity of ALR was tested by using isolated smooth muscle preparations (jejunum strips). The use of isolated tissues permitted us to examine the direct intestinal actions of ALR in the absence of bio-transforming bacteria or metabolic organs. In this study, the experimental models of the spontaneous, ACh and KCl-induced smooth muscle contractions were established. These models were conventional analytical methods of the spasmolytic activity and mechanisms of action of drugs or plant extracts (Moazedi et al. 2007). As the results shown in Figure 2, a graded relaxation of spontaneous contractions of rat-isolated jejunum was induced by ALR. The results may explain the deceleration of intestinal propulsion found *in vivo* study. The observations also indicated that ALR was biologically active and did not require bioactivation by intestinal flora or metabolic processes. Moreover, ALR was capable to relax the abnormal contractions on rat-isolated jejunum induced by ACh (10^−5^ M) and KCl (60 mM) (Figure 3). Accordingly, we speculated that the regulatory effects of ALR on GIM were mediated via the neural factors, as well as the myogenic ones.

ACh is regarded as an important neurotransmitter regulated the GIM (Bolton et al. 2008). The predominant muscarinic receptor subtypes present in smooth muscles throughout mammalian GI tracts are *M2* and *M3*. The two receptors mainly serve to produce excitations of GI smooth muscles by parasympathetic neurotransmitter ACh (Ehlert 2003). *M3* receptor is the main muscarinic receptors which mediated smooth muscle contractions in the GI tract. It causes intracellular Ca^2+^ mobilization through activation of phospholipase C (PLC) (Unno et al. 2006). The present study found that the contractions of rat-isolated jejunum induced by ACh were decreased by ALR. The results suggested the presence of cholinergic neurotransmitter release and receptor functions. Additionally, we concluded that the mechanism of ALR on *M3* postsynaptic receptors would be achieved through releasing intracellular Ca^2+^.

Ca^2+^ is a myogenic factor that plays an important role in smooth muscle cells contractions and relaxations. The contraction of smooth muscle preparations is dependent upon an increase in the cytoplasmic free Ca^2+^, which activates the contractile elements (Wang et al. 2006). Increase of intracellular Ca^2+^ concentration is mainly due to the calcium influx through voltage-dependent Ca^2+^ channels (VDCs) or release from intracellular stores in the sarcoplasmic reticulum. K^+^ at high concentrations (80 mM) can induce a depolarization via VDCs. High K^+^ (>30 mM) is also known to cause smooth muscle contractions through the opening of voltage-dependent l-type Ca^2+^ channels, thus allowing an influx of extracellular Ca^2+^ causing a contractile effect (Gilani et al. 2005; Soder and Petkov 2011). Based on the above researches, we used K^+^ at high concentration (60 mM) to induce smooth muscle contractions in this study. We found that the KCl-induced contractions of rat-isolated jejunum were effectively inhibited by ALR in a concentration-dependent manner. Substances causing the inhibitory action on high K^+^-induced contractions are considered to be blockers of calcium influx (Gilani et al. 2007). It suggested that the actions of ALR on relaxation were possible referred to the blockade of Ca^2+^ channels. To confirm the calcium channel blockade, the effects of ALR on CaCl_2_-induced contractions were determined. As the results shown in Figure 4, the CaCl_2_ concentration-dependent curves were shifted to the right in a noncompetitive manner after ALR treatment. The curve of CaCl_2_ concentration–response in the presence of ALR at the concentration of 1.6 mg/mL was similar to that of verapamil (a traditional calcium channel blocker) (Grasa et al. 2004). These results indicated that ALR acted as a calcium channel blocker interfering with Ca^2+^ movement to present the spasmolytic activity.

Furthermore, we explored if the antispasmodic activity displayed by ALR was mediated by gasotransmitters. Therefore, the effects of ALR on pretreatment with l-NAME (10^−4^ M, an inhibitor of nitric oxide synthase (NOS)) and PAG (10^−5^ M, an inhibitor of cystathionine-γ-lyase) of rat-isolated jejunum were tested. The results showed that relaxation curves of ALR were significantly displaced to the right pretreatment with l-NAME (*p* < 0.01), but not modified by PAG (*p* > 0.05). NO acts as a signalling molecule in vascular and GI smooth muscle. It is a vital gasotransmitter in numerous physiological and inflammatory processes (Moncada et al. 1991). Since a series of studies have demonstrated that NOS is present in the myenteric plexus, NO has become a most likely candidate for mediating nonadrenergic–noncholinergic smooth muscle relaxation through the GI tract (Ragy and Elbassuoni 2012). Endogenous NO from the smooth muscle is capable of regulating contractile tone. NO can activate soluble guanylyl cyclase (sGC) which increases the production of intracellular cyclic guanosine monophosphate (cGMP). The increase of cGMP results in the activation of protein kinase G that suppresses calcium influx and reduces the sensitivity of contractile elements to calcium (Ventura-Martínez et al. 2011). All these findings clearly revealed that the relaxation evoked by ALR was mediated by NOS–NO–cGMP signalling pathway.

Interstitial cells of Cajal (ICCs) control GIM, which are the pacemaker cells of GI muscularis propria and are involved in the generation of primary electrical pacemaker activity. Kim et al. (2017) found that *Magnolia officinalis* bark extract depolarizes ICCs pacemaker potentials (PPs) through M2 and M3 receptors via internal and external Ca^2+^ regulation through G protein pathways *in vitro*. They also showed that Liriope Platyphylla Wang Et tang dose dependently depolarizes ICC PPTs through M3 receptors via external and internal Ca^2+^ regulation and via G protein-, a phosphoinositide 3-kinase (PI3K)-, a PLC- and a protein kinase C (PKC)-dependent pathways *in vitro*. Moreover, they showed that naringenin reduced the amplitudes of the PPs of ICCs in a cGMP/NO-dependent manner by activating Ca^2+^-sensitive K^+^ channel (Kim et al. 2016, 2017; Kim and Kim 2017).

So, we suspect that the spasmolytic effects on GIM by ALR is likely related to ICCs. Therefore, further study on the relationship between ALR and ICCs is required.

Furthermore, phytochemical investigation on the ALR revealed the presence of major active ingredients, including sesquiterpenes, flavonoids and 2-(2-phenylethyl) chromone. These compounds have been reported to exhibit a wide variety of biological effects. It has been demonstrated that some sesquiterpenes of ALR exert antispasmodic, anti-ulcer and anti-inflammatory activities, such as hinesol, nootkanone and champacol identified in this study (Yamahara et al. 1990). The antispasmodic effects of ALR are involved in antidiarrhoea by regulating the GIM. Another active compounds identified in ALR were flavonoids. Flavonoids have been reported to possess a wide variety of biological effects such as antioxidant, anti-inflammatory, antispasmodic and antidiarrhoea activities (Rodriguez-Mateos et al. 2014). All these suggested that the inhibitory actions of ALR *in vivo* and *in vitro* tests were probably attributed to the constituents of the spasmolytic and anti-inflammatory properties, especially for sesquiterpenes and flavonoids. The chemical analysis and the identification of active ingredients further supported the protective effects of ALR on the GIM.

## Conclusions

In conclusion, the present study suggested that the GI dynamic mechanism of ALR methanol extract was the main inhibitory activity. The antispasmodic activity of ALR was mediated through blockade of muscarinic receptors, Ca^2+^ channels and regulation of NOS–NO–cGMP pathway, but no participation of H_2_S. Besides, the effects of ALR on GIM were probably attributed to the spasmolytic and anti-inflammatory properties of active constituents, focusing on sesquiterpenes and flavonoids in ALR. Thus, the use of this medicinal plant in traditional medicine for the treatment of GIM diseases is supported and justified by the pharmacological properties described in this study.
